# Can Gait Features Help in Differentiating Parkinson’s Disease Medication States and Severity Levels? A Machine Learning Approach

**DOI:** 10.3390/s22249937

**Published:** 2022-12-16

**Authors:** Chariklia Chatzaki, Vasileios Skaramagkas, Zinovia Kefalopoulou, Nikolaos Tachos, Nicholas Kostikis, Foivos Kanellos, Eleftherios Triantafyllou, Elisabeth Chroni, Dimitrios I. Fotiadis, Manolis Tsiknakis

**Affiliations:** 1 Biomedical Informatics and eHealth Laboratory, Department of Electrical and Computer Engineering, Hellenic Mediterranean University, Estavromenos, 71410 Heraklion, Crete, Greece; 2 Computational BioMedicine Laboratory, Institute of Computer Science, Foundation for Research and Technology—Hellas, Vassilika Vouton, 71110 Heraklion, Crete, Greece; 3 Department of Neurology, Patras University Hospital, 26404 Patra, Greece; 4 Unit of Medical Technology and Intelligent Information Systems, Department of Materials Science and Engineering, University of Ioannina, 45110 Ioannina, Greece; 5 Biomedical Research Institute, Foundation for Research and Technology—Hellas, 45110 Ioannina, Greece; 6 PD Neurotechnology Ltd., 45500 Ioannina, Greece

**Keywords:** gait analysis, Parkinson’s disease, ON/OFF medication, MDS-UPDRS, severity levels, insoles, pressure sensors

## Abstract

Parkinson’s disease (PD) is one of the most prevalent neurological diseases, described by complex clinical phenotypes. The manifestations of PD include both motor and non-motor symptoms. We constituted an experimental protocol for the assessment of PD motor signs of lower extremities. Using a pair of sensor insoles, data were recorded from PD patients, Elderly and Adult groups. Assessment of PD patients has been performed by neurologists specialized in movement disorders using the Movement Disorder Society—Unified Parkinson’s Disease Rating Scale (MDS-UPDRS)-Part III: Motor Examination, on both ON and OFF medication states. Using as a reference point the quantified metrics of MDS-UPDRS-Part III, severity levels were explored by classifying normal, mild, moderate, and severe levels of PD. Elaborating the recorded gait data, 18 temporal and spatial characteristics have been extracted. Subsequently, feature selection techniques were applied to reveal the dominant features to be used for four classification tasks. Specifically, for identifying relations between the spatial and temporal gait features on: PD and non-PD groups; PD, Elderly and Adults groups; PD and ON/OFF medication states; MDS-UPDRS: Part III and PD severity levels. AdaBoost, Extra Trees, and Random Forest classifiers, were trained and tested. Results showed a recognition accuracy of 88%, 73% and 81% for, the PD and non-PD groups, PD-related medication states, and PD severity levels relevant to MDS-UPDRS: Part III ratings, respectively.

## 1. Introduction

Parkinson’s Disease (PD) is one of the most prevalent neurological diseases. The global prevalence of PD patients is expected to reach 12 million cases by 2040 [1]. The pathophysiology of PD is extensively researched and has been linked with genetic and environmental risk factors [2]. The main neuropathological hallmarks of PD include neuroinflammation, degeneration of dopaminergic neurons in the substantia nigra pars compacta, and accumulation of misfolded α-synuclein proteins as intra-cytoplasmic Lewy bodies and neurites [3,4]. The manifestations of PD include both motor and non-motor symptoms. The cardinal motor symptoms of PD are bradykinesia, rigidity, tremor, and postural instability [2,5], whilst the non-motor symptoms include depression, cognitive decline and dementia, sleep disorders, dysphagia, constipation, urinary dysfunction, and orthostatic hypotension, among others [5,6,7,8]. At the moment, there is no cure for PD, however, the therapeutic process includes among others the admission of drugs for symptomatic relief, mainly focusing on dopamine replacement strategies [9]. Based on patient’s clinical characteristics, treatment may include surgical approaches, such as deep brain stimulation (DBS) [10]. The diagnosis of Parkinson’s disease is, until today, mainly based on the clinical assessment of the patient [11]. The Movement Disorder Society—Unified Parkinson’s Disease Rating Scale (MDS-UPDRS) is considered the gold standard for the assessment and monitoring of Parkinson’s disease [12], followed by the well-established Hoehn and Yahr (HY) scale [13].

Gait analysis is critical for managing and monitoring the progression of PD symptoms. Wearable sensor-enabled systems have been developed to extract numerous aspects of PD motor symptoms, such as gait abnormalities, imbalance problems and tremor, which cannot be derived merely from the clinical picture of the patient [14]. There is evidence that gait patterns differentiate between PD patients and healthy controls [15,16]. There has been a surge of interest in using machine learning and deep learning approaches that utilize sensor signals (i.e., accelerometers, gyroscopes, pressure sensors) and/or videos to monitor and forecast PD progression [14,17,18,19,20,21,22,23,24]. However, video-based motion analysis systems require expensive equipment and are limited in equipped-indoor environments, while wearable sensors are a more affordable solution, providing a method for long-term monitoring under real-life conditions [25,26,27,28]. These research results demonstrate promising potential for the deployment of systems for the monitoring and management of PD, based on sensor signals and machine-learning methods. Although a lot of studies focus on motor symptoms, and specifically, gait analysis, and there are standardized assessment tests, we observe that a comprehensive protocol is lacking, i.e., the studies are exploring only one type of test, they include walking at normal pace only, they often use unbalanced datasets, and in several cases a control group is lacking. Another important aspect that needs to be considered is the need for the parallel assessment of PD participants by specialized clinicians using state-of-the-art quantification metrics, such as the MDS-UPDRS [12]. These issues are also observed, in full or in part, in the limited number of publicly available datasets that include sensor-based gait data and focus on PD patients, as shown in Table 1. Specifically, the Gait in Parkinson’s Disease dataset [29], includes the vertical ground reaction force records of a large number of participants (93 PD patients and 73 healthy controls) along with demographic information and scores of PD scales (the H and Y staging and/or the UPDRS), however only self-selected pace of walking for 2 min was recorded, with a subset of participants to also perform a dual tasking test, while ON/OFF medications states were not addressed. The Daphnet Freezing of Gait Data Set [30], encompass 3D acceleration data derived from sensors on lower extremities, while participants performed three different tests elaborating the activities of walking straight with 180° turn, random walking with stops and 360° turns and, simulating activities of daily living, while participants’ performance has been rated with the use of H and Y scale. However, only 10 PD patients (who present the FoG symptom) were recorded, there was no control group, ON/OFF medication states were not addressed, and only self-selected pace of walking was examined. Finally, The Smart-Insole Dataset [16], includes data derived from pressure sensor insoles, while participants performed two different sets of tests elaborating different walking speeds, however the performance of the participants has been rated using four items of the MDS-UPDRS, the dataset is small (8 PD patients, 9 elderly and 13 adults are included), while ON/OFF medications states were not addressed. These issues introduce limitations and make the comparative analysis of results very difficult, if not impossible. Moreover, the assessment of PD patients in ON and OFF medication states has been highlighted as an aspect that needs further investigation [31]. Finally, for the accurate automated detection of PD patients’ mild-stage motor symptoms, it is important to classify the different severity levels of the disease using as a reference point the quantified metrics that are applied in clinical practice.

In such a context, the present work contributes to the challenging task of PD monitoring, by introducing a computational pipeline for identifying relations between the spatial and temporal gait features, PD-related medication states and disease severity levels, as well as for separating PD patients from non-PD groups. To this end, we elaborate on a comprehensive experimental protocol for the assessment of PD motor signs of the lower extremities, as presented in Section 2. The protocol is based on literature findings regarding the most popular tests employed in the domain, and on the description of relevant items of MDS-UPDRS-Part III: Motor Examination, while it encompasses the assessment of different walking speeds. Recordings have been obtained from PD patients, and two control groups, elderly and adults. For PD patients, both, ON and OFF medication states have been examined. Clinical assessment has been performed by neurologists specialized in movement disorders, utilizing the MDS-UPDRS-Part III: Motor Examination. Gait-related spatial and temporal characteristics were extracted from the raw pressure sensor data, and feature analysis techniques have been deployed, as described in Section 3. Furthermore, a set of machine learning algorithms were explored for the classification of the different groups of participants (PD, Elderly, Adults); ON and OFF medication states; and diseases severity levels based on the MDS-UPDRS: Part III ratings, the results of which are presented in Section 4.

## 2. Methodology

For the needs of this study, 44 participants, including PD patients, Elderly, and Adults, performed the Smart-Insole Gait Assessment Protocol, while wearing a pair of pressure sensor insoles. Informed consent was obtained from all subjects involved in the study. PD Patients were recruited from the Movement Disorders Clinic, Patras University Hospital, Greece, while the Elderly and Adults were enrolled at the Hellenic Mediterranean University, Crete, Greece. The Smart-Insole Gait Assessment Protocol has received ethical approval from the Patras University Hospital Research Ethics Committee (Approval number: 279/14.05.2021) and the Hellenic Mediterranean University Research Ethics Committee (Approval number: 9/01.04.2020).

### 2.1. Materials and Setup

**Figure 1:**
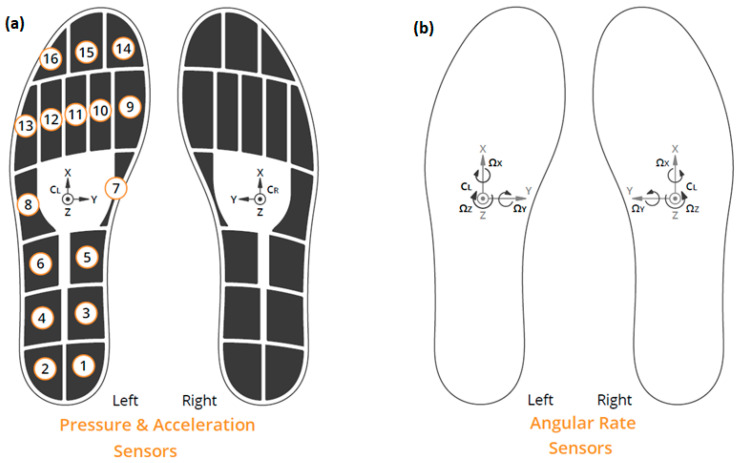
The Moticon Science Sensor Insole (Μodel Ιnsole 3). (**a**), shows the arrangement of the 16 pressure sensors and the positioning of the 3-axis acceleration sensor. (**b**) shows the positioning of the angular rate sensors. Reproduced with permission from Moticon ReGo AG [35].

For data acquisition, a validated sensor insole system [16,32,33,34], namely, the Moticon SCIENCE [35], was used. The insole system, as shown in Figure 1, incorporates 16 capacitive type pressure sensors, a 6-Axis Inertial Measurement Unit (IMU) sensor for acceleration and angular rate data, while it includes the units of power supply, storage, and data transmission into the insole. For the recordings, a sampling rate 100 Hz was employed. Each generated file includes the timestamp (ms) among with, the pressure of the 16 sensors (N/cm^2^), the acceleration in the x, y, z axes (g), the angular rate in ωx, ωy, ωz (dps), and the computed by Moticon, total force (N) and center of pressure in the x, y coordinates. To avoid inconsistencies in the material setup, lightweight and flexible pairs of shoes were purchased in which the insoles were fitted. The participant’s performance was captured with the use of two cameras placed at the middle and end of the inquest route.

### 2.2. A Protocol for the Assessment of Gait of Parkinson’s Disease Patients Using Wearable Sensors

As previously stated, the Smart-Insole Gait Assessment Protocol for PD patients was defined. The protocol focuses on the exploitation of bradykinesia and postural instability, from the cardinal features of PD, and the use of an additional motor symptom that is extensively studied because of its association with disease severity and increased risk of falls, i.e., Freezing of Gait (FoG) [36].

In the majority of studies, the tests performed for the assessment of gait characteristics of PD patients, are: (1) Free Walking for a predefined duration of time and at a normal pace of walking; (2) the Timed Up and Go test (TUG test), where the participants rise from a sitting position, walk a 3 m distance, turn, walk back and sit on a chair; (3) walking in a corridor with obstacles, and; (4) the Dual-Task test, where the participants walk and at the same time undertake a second process, such as an arithmetic operation, conversation, transferring an object, etc. [37,38,39,40,41,42,43,44]. For movement disorders experts, the MDS-UPDRS and particularly Part III: Motor Examination is considered the gold standard for clinical assessment.

Therefore, in developing our research protocol, we have taken into consideration literature findings and guidance of clinical experts and focused on six items of the MDS-UPDRS-Part III: Motor Examination that involve lower extremities, namely 3.9-Arising from chair, 3.10-Gait, 3.11-Freezing of gait, 3.12-Postural Stability, 3.13-Posture, 3.14-Global spontaneity of movement. We also introduced a test for assessing FoG based on the findings of previously published work [45]. As a result, the Smart-Insole Gait Assessment Protocol consists of a set of five different tests, namely, the Walk Straight and Turn test, a modified version of the Timed Up and Go test, the Static Balance test, the Retropulsion test, and finally, the FoG and Dual Tasking test. These are described in detail in Section 2.2.1, Section 2.2.2, Section 2.2.3, Section 2.2.4 and Section 2.2.5. Participants were asked to complete the tests as a continuous set of movements.

#### 2.2.1. The Walk Straight and Turn Test

**Figure 2:**
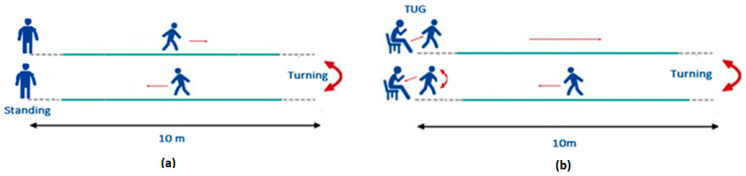
The Walking Straight Test Left figure (**a**), and The Timed Up and Go Test right figure (**b**).

The first test of the protocol is the Walk Straight and Turn (WST) test in which participants, starting from a standing position, walk in a straight route for 10 m, then turn back and return to their starting position (Figure 2a). The WST Test is organized in line with the description of the MDS-UPDRS item 3.10-Gait and contributes to the rating of items 3.11-Freezing of Gait and 3.13-Posture, whilst in parallel, it provides valuable information for the estimation of stride amplitude and speed, as well as heel strike and turning. The WST test is performed two times at slow, normal, and fast walking speeds, as perceived by each participant. The importance of exploring the gait characteristics at different walking speeds, especially for PD patients, has been revealed in several published studies [16,46,47]. Gait analysis on both slow and high speeds has shown that gait characteristics are significantly affected and can provide valuable information for differencing gait between different groups of participants and on PD patients with mild and moderate severity.

#### 2.2.2. The Modified Timed Up and Go Test

The typical form of TUG test requires participants to rise from a sitting position, walk a 3 m distance, turn around and walk back to sit on the chair, while they are rated with a completion time score [38]. The modified Timed Up and Go test (mTUG), shown in Figure 2b, is configured exactly as the MDS-UPDRS item 3.9-Arising from a chair, for its initial phase, where participants cross their arms across their chest and then stand up. Once participants have managed to stand up, they are requested to walk a 10-m route, turn around, walk back, and return to their sitting position. The test is performed twice, at a normal pace of gait.

#### 2.2.3. The Static Balance Test

**Figure 3:**
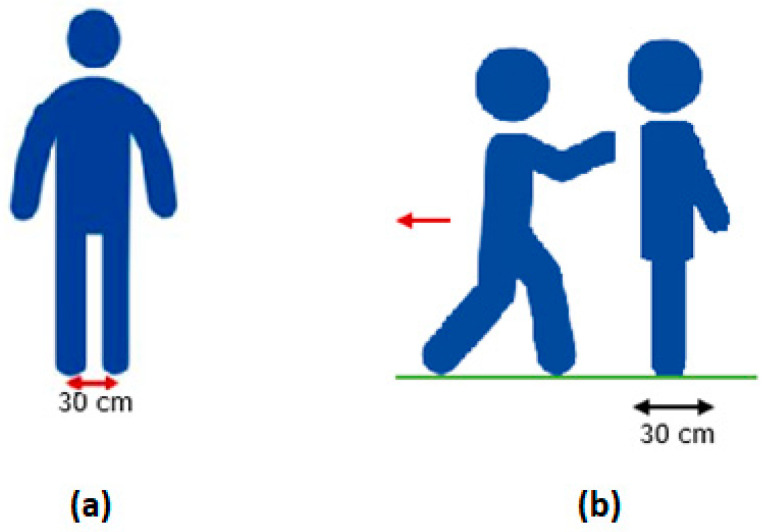
The modified Timed Up and Go test—Left figure (**a**), and The Static Balance test—right figure (**b**).

For the Static Balance test, participants are requested to stand at an upright position with their feet facing forward, at a marked distance of 30 cm, and looking straight ahead (Figure 3a). Participants are instructed to stand motionless for 10 s with their eyes open and then to repeat the test with their eyes closed for another 10 s. The pressure signals collected in this test provide information concerning the center of pressure at a static position.

#### 2.2.4. The Retropulsion Test

The Retropulsion test, also known as pull test, is a commonly used test in clinical practice for assessing postural stability in PD patients. The test is configured in line with the description of the MDS-UPDRS item 3.12-Postural Stability. Participants place themselves in the position of the Static Balance test, while the instructor, after informing the participant what is about to happen, quickly and forcefully pulls participants from their shoulders backward, to displace their center of gravity, so that they are forced to undertake steps backward to retain their position (Figure 3b).

#### 2.2.5. The FoG and Dual Tasking Test

**Figure 4:**
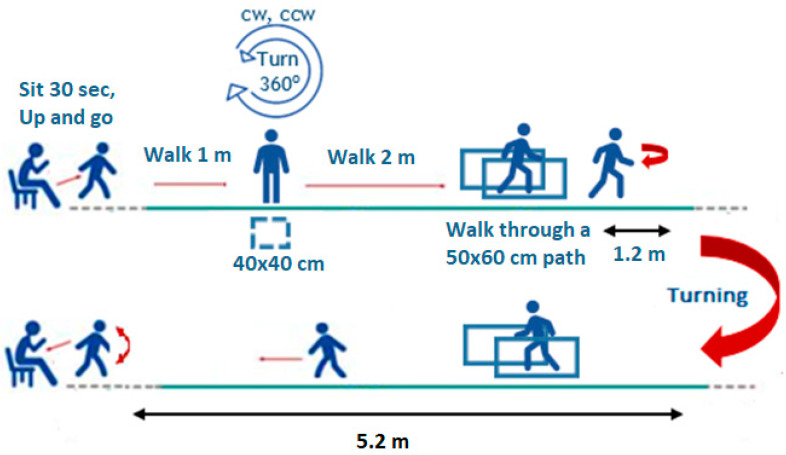
The FoG and Dual Tasking test based on [45].

The FoG and Dual Tasking test is in line with a previously published work by Ziegler et al. [45], for the assessment of FoG and motor and mental Dual Tasking. For this test, as shown in Figure 4, participants are requested to sit and relax in a chair for 30 s, then stand up and walk for 1 m till they place themselves in a 40 cm × 40 cm square marked on the floor, where they need to perform two, in-position, turns of 360°, one clockwise and one counter-clockwise; then, they walk for 2 m and walk through a narrow passage of 50 cm × 60 cm (created with the use of two chairs); once they walk out of the passage they are requested to turn around (given a 1.2 m available space for turning); walk back through the narrow passage and walk in a straight route until they are in a position to sit back in the chair. This test is performed in three different versions: (a) the basic test, including only the walking route as described; (b) the motor-dual tasking, where they perform the walking route and at the same time hold a glass full of water in their hand; and (c) the mental-dual tasking, where they perform the walking route and at the same time perform loudly serial deductions of sevens starting at 100. The test is performed at a normal pace of walking as perceived by each participant.

### 2.3. Participants

In this study, 44 participants in total were included (Table 2), belonging to three different groups: Adults, Elderly with no musculoskeletal or neurological diseases that could affect their gait or balance, and PD patients; with age-related inclusion criteria 20–59, above 60, and 20 years, respectively. PD participants were enrolled in both ON and OFF medications states. Particularly, for each PD participant OFF state was recorded early in the morning, while the ON state was recorded one hour after they had received their medication, on the same day. Two PD participants were on a dopamine-continuous infusion pump (D-CIP) and thus only ON states were recorded.

### 2.4. Parkinson’s Disease Ratings: MDS-UPDRS/Subsets and Severity Levels

The Part III: Motor Examination of the MDS-UPDRS [12] was applied for the rating of PD participants, on both ON and OFF states, by neurologists specialized in movement disorders who actively participated in the recordings. The MDS-UPDRS-Part III: Motor Examination, consists of 33 items with a maximum total score of 132, which indicates the most severe disease state. The gait performance of the Adults and Elderly participants was evaluated by the neurologists after examining video recordings (as in our previous work [16]) and rated on a subset of six items of the MDS-UPDRS-Part III: Motor Examination that involve lower extremities, namely 3.9 -Arising from a chair, 3.10-Gait, 3.11-Freezing of gait, 3.12-Postural Stability, 3.13-Posture, and 3.14- Global spontaneity of movement (named as Control Subset). The maximum total score of Control Subset is 24. The details of participants and their scores at the relevant subsets are presented in Table 3.

The demanding task of early detection of Parkinson’s disease presents various challenges and is highly dependent on the severity of the disease and the expert’s experience. For the early diagnosis of PD, accurate detection of symptoms is required whilst the disease is at a mild severity stage. Computationally, this needs not only to detect the presence of symptoms but also to perform a multiclass classification at different severity levels.

In the present work, we focus on the estimation of PD severity levels by calculating the statistical significance of the computed gait characteristic, as proposed by Martínez-Martín et al. [48] based on the ratings of part III of the MDS-UPDRS. Particularly, four severity levels were explored in relation with the MDS-UPDRS-Part III ratings: normal (0–8), mild (9–32), moderate (33–58) and, severe (59–132), [14,17,49].

## 3. Data Analysis

Gait analysis necessitates a quantitative examination of force factors, as well as time and distance parameters, through the calculation of significant features (temporal and spatial). Temporal characteristics can be further analyzed as pure temporal, phase temporal, or spatiotemporal. In our study, the phase-temporal characteristics have been normalized based on the duration of the gait cycle, while the spatial characteristics were determined by taking into consideration the overall distance travelled in each test. The mathematical formulas used for the estimation of the gait temporal and spatial characteristics are described in detail in [16]. Prior to the analysis of the recorded data, precise annotation of data regarding the gait cycle events, namely Heel Strike-Foot Flat-Heel Rise-Toe Off-Heel Strike, was performed. We employed a hybrid model of automated labeling and manual cross-checks using the signals and videos captured. For the automated annotation of gait events, we adopted a previously developed gait event detection algorithm which is based on pressure parameters and transition states of gait characteristic events, as reported in [16].

### 3.1. Gait Features Extraction

In the present work, we exploit data from the signal segment that corresponds to the straight-line walking of the mTUG test and the WST test, when the latter is executed in Slow, Normal and High speeds. Specifically, two recordings per test were analyzed for each participant. The distance of straight-line walking is 10 m ± the distance that corresponds to one step, since some participants turned slightly before or slightly after the marked 10 m aisle. This fluctuation has a small influence on the estimated values of the spatial features when the range of step counts (14.35–22.48) is considered (see Table 4). To verify this, we performed a cross-check by adding or subtracting one step, on our calculations. We observe that such a fluctuation results in modifications to the estimations restricted to the second decimal place.

The estimated temporal and spatial gait characteristics (18 in total) are presented in Table 4 for the different participants groups, i.e., Adults (S), Elderly (EL), PD patients (PD). Table 5 shows the relevant features for the PD group of participants in relation to the ON and OFF medication states, whereas Table 6 presents those for the PD group of participants relative to their Severity Level, estimated using the MDS-UPDRS: Part III. The results are presented as mean values together with standard deviation, rounded to two decimal places.

For the analysis of the collected data, we firstly extracted and examined the spatial and temporal gait characteristics of all different participant groups on WST-Slow, Normal, High and the mTUG tests, as shown in Table 4. Participants were instructed to perform both the WST-Normal and the mTUG tests using a normal walking rate. However, we retained the tests’ data separately so that it would be feasible to conduct future comparative studies and to examine the effect of prior physical activity that is noted in the mTUG test (rising from a chair with arms crossed on the chest).

Normal gait speed, in terms of comfort, has been reported to be approximately 1.30 m/s for adults of 60 years [50], approximately 1.20 m/s and 0.95 m/s for the elderly aged between 70–79 years and 80–99 years, respectively [51], while for PD patients with a mean age of 65 years, normal gate speed has been reported to be approximately 0.95 m/s [52]. In our work, the control groups (S and EL), and PD group both have a mean age of 60 years, and we recorded an average gait velocity of approximately 1.22 m/s and 0.97 m/s, respectively. In the mTUG test, we observed an increase in gait velocity for all groups, although they have been instructed to employ a normal pace of walking (as in WST-Normal test).

Examination of the estimated features on the different groups and tests shows that different speeds of gait affect the results. Particularly, for all groups of participants, as we move from WST-Slow to Normal and to High tests, and therefore the gait velocity of participants increases, we observe that all temporal characteristics decrease, while the swing phase (%) and the spatial characteristics increase (Table 4). We observe a similar behavior when the results from WST-Slow to the mTUG tests are examined.

The Walk Ratio is excluded from this observation. The Walk Ratio is known to be constant at approximately 6.5 ± 0.8 (mm/step/min) for healthy adults and for those with pathological gait should decrease [53]. This is indeed observed in our results since the PD group has lower values when compared to those of the control groups, in all different tests. Regarding Walk Ratio, literature findings suggest that when comparing results from normal to slow walking speeds, increased values are expected, something that was also observed for all groups of participants when examining the mTUG to WST-Slow test, while when examining WST- Normal to Slow test it is noted only in the case of EL group (for S and PD groups values are concentrated around the same level), possibly due to the smaller differences of gait velocity between different tests, below 0.3 m/s [54,55]. Moreover, in each test, PD patients had the lowest results regarding step and stride length, gait velocity, and step frequency while they had the highest results on the number of steps. The percentages of the stance and swing phases, which in a normal gait cycle are expected to be 60% and 40%, respectively, are met in most cases with a slight deviation from 0.5–1.5%. All of these observations are in line with literature findings [31,54,55,56,57]. 

Subsequently, in order to examine the effect of ON and OFF medication states on the performance of PD participants regarding their spatial and temporal gait characteristics, we have extracted gait-related features for both medication states, as shown in Table 6. In general, the behavior of the features exploring the different walking speeds is in line with the previously described findings shown in Table 4. Regarding the ON and OFF phases, we observe an increase of values in steps number, double support (%), and step frequency features, and a decrease in step and stride duration, swing time, single support (%) and Walk Ratio, features, in ON phase. Gait velocity does not appear to have a notable effect in the ON phase, for all tests. Step and stride length slightly decrease in the WST-Normal and High tests for the ON phase, while they remain at the same level in WST-Slow and mTUG tests.

Subsequently, we separated the PD participants according to their disease severity levels based on their ratings on MDS-UPDRS-Part III, by applying the cut-off values described in Section 2.4; the relevant results are presented in Table 6. We should point out that there are no recordings in the severe level as rated by the MDS-UPDRS-Part III. Although we did record a PD patient (PD015) classified as severe PD, we had to exclude the recordings because he needed support to perform the WST-High and the mTUG tests and therefore his weight distribution and pressure factors were affected. Examining the results for normal pace of walking, from mild to moderate levels we observe that as the severity of the disease increases, single support (%), swing phase (%), Walk Ratio, spatial and spatiotemporal characteristics decrease, while all the rest increase. These observations are also met in the case of WST-Slow with the difference that swing time decreases. For WST-High, features behavior differentiate from WST-Normal observations in moderate severity with the swing time, single support (%), and swing phase (%) to increase, while with the double support time, the double support (%), and the stance phase (%) to decrease. Regarding Walk Ratio, we observe that on moderate severity levels it is, in all tests, decreased (spanning from 5.09 ± 1.17 to 5.41 ± 1.39), which agrees with literature findings in pathological gait, 5.36 ± 0.86, Ref. [53], in comparison with mild severity levels (spanning from 6.63 ± 1.62 to 6.78 ± 1.43), which overlaps with literature on normal gait of healthy adults (6.5 ± 0.8) [53]. Therefore, the results indicate that as the severity level increases, the Walk Ratio decreases, however, for mild stages of the disease it may not be indicative.

In what follows we focus on feature selection techniques to reveal the dominant features for model training, which are described in Section 3.2; the results of which are presented in Section 4. In so doing, we also take into consideration literature findings regarding the importance of a deeper study of the ON/OFF medication states and disease severity levels [17,31,48].

### 3.2. Feature Selection and Model Training

In the context of our computational pipeline, feature selection techniques were applied to reveal the dominant features to be used for model training. We focus our analysis on identifying relations between the spatial and temporal gait features, the PD-related medication states, and disease severity levels. Specifically, we focused on four classification tasks. We initially focused on separating PD patients from non-PD patients in a binary classification problem. Non-PD patients are the elderly and adult subjects in our recordings. We then split the elderly and adults in a three-class classification problem, i.e., PD patients, elderly, and adult participants. The next classification problem focused on differentiating PD patients based on their medication state, the ON and OFF states. Finally, we focused on the MDS-UPDRS Part III ratings and the relevant PD severity levels, i.e., normal, mild, moderate, and severe, as described in detail in Section 2.4. However, our recordings include data from PD patients in only two severity levels, i.e., mild, and moderate. Although we did record a patient rated at the severe level, we had to exclude his recordings since he needed support to complete all of the tests and therefore this would induce bias.

Feature-wise normalization was conducted prior to model fitting to address the possible issue of biased behavior, which result from uneven contribution of variables measured on different scales to the model-fitting and model-learned functions [58]. After extracting the gait data, we scaled each feature to a range of 0 to 1 using the MinMaxScaler function. In addition, we created a correlation matrix displaying the correlation coefficients between the variables as a preliminary data interpretation tool and as input for a more sophisticated investigation a Linear Mixed Model Analysis. The input data for each classification approach are the most significant features, selected among the 18 extracted gait temporal and spatial features based on the correlation matrix and the LMM analysis, presented in Section 4.1.

Considering the relevant literature and the size and properties of our dataset, we selected three well-known and widely used classifiers to explore their performance. More specifically, AdaBoost (AB), Extra Trees (ET), and Random Forest (RF) classifiers were trained and tested during the classification procedure [59]. To fine-tune the hyperparameters of each classifier we performed a GridSearch iterating 1000 times through the training data, to find the combination of parameters that maximizes the overall performance and accuracy. Finally, we split the data into training and testing, with the number of the test data being 20% of the total number of examples, according to the Pareto principle [60]. We first train our model on the training set and then we use the data from the testing set to measure the accuracy of the resulting model. It is important to mention that we took into consideration that the test set is large enough to yield statistically meaningful results, and that it is representative of the dataset. We evaluated the models using the metric of accuracy. Furthermore, we validated the models using a k-fold cross-validation (k = 5). Choosing the right k-value is important as it can affect the level of accuracy, variance, and bias of a model. According to the literature [61], choosing a k-value of 5 or 10 has proven to provide a low bias and a modest variance. The larger the value of k becomes it becomes more computationally impractical, therefore in our work we chose k = 5 as more computationally efficient. The models were built using Python (3.8 Python packages, version numpy 1.17.2, sklearn 0.21.3, pandas 0.25.1).

## 4. Results

In this section, the results of the Statistical analysis and the Machine Learning analysis, which have been performed seeking to identify relations between gait parameters and (a) PD vs. non-PD groups, and (b) PD-related medication states, and severity levels, are presented.

### 4.1. Statistical Analysis

Our dataset consists of dependent observations since there are multiple samples of the same participant in the same group and/or medication state and/or severity level. Thus, an analysis that assumes independent observations in each class or between the classes themselves, such as ANOVA, cannot be employed. To determine which gait features are affected by the existence of the disease, the medication state or the severity level of PD, a Mixed Linear Model (MLM) analysis with Bonferroni adjustment, and with the disease group (Adults, Elderly, and PD patients), medication state or MDS-UPDRS:Part III, as a fixed factor, and participant ID as a random factor was employed for each test. The level of statistical significance was set at *p* < 0.05. We present the results of MLM analysis regarding the statistical significance of features derived from slow, normal, and high-pace walking (elaborating the WST Slow, Normal, High tests and mTUG), to explore the effect of gait velocity in differentiating gait patterns.

The mixed linear model analysis revealed significant correlations of features between ON/OFF medication states and between severity levels based on the MDS-UPDRS-Part III Total Score, as shown in Table 7, for each different type of test. The results indicate the importance of using different gait velocities since different features are identified as being significant for each type of test. This allows for not only examining the behavior of features in relation to different gait velocities but also comparing results with the literature by exploiting a range of velocities, as the normal pace of walking may differ significantly per participant.

### 4.2. Machine Learning Analysis

We also focused on identifying relations between gait features and a participant’s group (PD and non-PD, EL, S) or the medication state (ON/OFF). Moreover, a machine learning analysis was performed based on the severity levels as denoted by the MDS-UPDRS Part III Total Score. The results of the classification studies using data derived from the Smart-Insole Gait Assessment Protocol for PD patients are presented in Table 8.

#### 4.2.1. Participant Groups Classification, PD/non-PD and PD/EL/S

An initial and necessary classification experiment is that of distinguishing PD participants from non-PD. To do so, the EL and S groups of participants were clustered into one, namely non-PD, to test the ability of the machine learning model. The results show that the RF algorithm, when exploiting derived data from the WST Slow test, recognizes PD patients from non-PD with an accuracy reaching 88%, as shown in Table 8. Moreover, high accuracy rates are also found when exploiting the mTUG and WST High tests, with AB providing 85% and 83% accuracy, respectively. However, in the case of the WST Normal test the accuracy rates are lower, at 73%. These results, verify once more the need to employ different walking speeds when it comes to gait analysis for PD patients. Overall, the AB and RF algorithms appear to be the best-performing.

Following the binary classification, we sought to perform a three-class classification, i.e., PD, EL, and S. As presented in Table 8, the classification accuracy reached 77%, when using the RF classifier in the WST-Slow. In the WST-Normal, High and the mTUG tests, the classification accuracy reached 64%, 70%, and 66%, respectively. The reduction in the classification accuracy in comparison to the binary classification is expected since recognizing PD from EL can be challenging due to possible pathology in gait caused by aging in the control group EL, and mild severity of disease in the PD group. Therefore, the results are encouraging, demonstrating the ability of the gait features to discriminate between the three classes PD, EL and S.

#### 4.2.2. Classification between Medication States ON/OFF

Focusing on PD patients, we moved on in attempting to distinguish PD patients based on their medication state, i.e., ON and OFF classes. The results obtained are shown in Table 8. The highest classification accuracy is achieved during the mTUG test with an accuracy of 73%. Concerning the algorithms, we observe that as the walking speed decreases, the success rate in the predictions of the algorithms also decreases, providing evidence that the drug effect is more easily observed when the participants walk at a higher speed. In addition, ET is the best performing classifier in three of the total four tests. Based on the results, we also observe that in the mTUG and WST-High tests, the difference between the OFF and ON conditions is more noticeable. This may be because the mTUG and WST-High tests require more vigorous physical activity and therefore cause more fatigue to the patient compared to the other tests. Specifically, the mTUG test involves standing up with no support from upper limbs and sitting on a chair, while the WST-High test involves walking at a fast pace. Therefore, the lack of medication (i.e., OFF state) and the fatigue of the lower limb muscles further intensify the gait symptoms of the disease during the conduction of WST-High and mTUG tests.

#### 4.2.3. Parkinson’s Disease Severity Levels Based on MDS-UPDRS: Part III Ratings

The ability of machine learning algorithms to classify the PD Severity Levels based on MDS-UPDRS:Part III ratings are presented in Table 8. Parkinson’s disease among the severity levels of mild and moderate was exploited. The results indicate successful prediction rates of 81% and 77% for the mTUG and WST-Slow tests, respectively. In both cases, AB appears as the best performing algorithm. Rates close to 73% are also observed in the case of the RF algorithm.

## 5. Discussion

In this work, we exploited pressure sensor insole data, derived from three groups of participants (Adults (S), Elderly (EL), and PD patients) performing the Smart-Insole Gait Assessment Protocol for PD patients. We have extracted several spatial and temporal features and employed feature selection methods to identify the most significant. A variety of estimators were tested in attempts to separate PD patients from healthy controls and to distinguish between PD patients’ ON/OFF medication states. Finally, we investigated the possibility of discriminating between the severity levels of PD, derived from MDS-UPDRS: Part III Total Score. Machine learning models were trained using gait features, while three classification algorithms were tested, seeking to identify those with the highest success rates.

Our statistical analysis shows that various gait extracted features can be used to differentiate the medication state of PD patients as well as the severity levels of the disease (Table 7). To differentiate the medication status (ON/OFF) proved to be the most challenging. Based on our findings, the ON/OFF medication state of PD patients is associated with a variety of different gait features for each test. Notably, the Swing Time is significantly affected by the medication state in all tests. Moreover, the Step Number and the Single Support Time significantly correlate in the WST- Slow and High tests, while Step Frequency and Walk Ratio significantly correlate in the WST- Slow and Normal tests. Apart from this comparison, a number of gait features showed the potential to be useful indicators of PD severity level, based on MDS-UPDRS Part III. Generally, it appears that Swing Time (s), Single Support (%), Stance Phase (%), and Swing Phase (%) are the gait-related features that are highly affected by the severity levels of the disease.

From the results presented in Table 8, the highest performance was observed during the binary classification between PD and non-PD participants, achieving 88% by employing the RF classifier during the WST-Slow test. However, splitting the “non-PD” class into healthy elderly and adult subjects proved difficult, resulting in a considerable reduction of the model’s performance, achieving a 77% accuracy with RF classifier during WST-Slow test. In terms of predicting the medication state of PD patients, the highest performances were observed during the mTUG and WST-High tests, with up to 73% correct predictions when using the ET estimator. This finding confirms the results of the statistical analysis, as these two tests require more effort from the participant and therefore cause greater physical fatigue. Consequently, by observing the results in the test requiring a higher physical stress, it is evident that the administration of dopamine drug enhances muscular movements and durability during walking. Regarding the identification of severity levels based on the MDS-UPDRS: Part III ratings, a prediction rate of 81% was observed in the mTUG test and 77% in the WST-Slow test, using the AB algorithm.

The analysis of both ON and OFF medication states is crucial to fully describe PD patient’s performance but also to study the effect of levodopa which relates patients’ responsiveness to a large number of factors including patients PD type and PD severity level [62,63]. Moreover, contradictory findings may be found in the literature regarding features association with gait velocity, possibly due to the fact that the notion of “normal gait velocity” is strongly subjective and a variety of features are pace-related [31,54,55]. Our findings indicate the presence of different dominant features in different walking speeds (see Table 7), which is in line with literature findings indicating that the effect of the ON phase in the spatial and temporal features is mediated by self-selected gait velocity [64]. Therefore, these findings support our claim that recordings of slow, normal, and high walking speeds, on both the ON and OFF phases of a patient, are required for a comprehensive analysis, also exploring in parallel the severity levels of the disease.

Therefore, all the challenges that have been earlier identified need to be addressed for an in-depth approach on PD gait analysis and even more so for designing an accurate gait-based PD monitoring system. However, there is a limitation in the confidence of the estimations obtained when only gait analysis is explored. We reaffirm recent findings, [65,66], that a more robust approach should include the integration of motor symptoms from lower and upper extremities and other non-motor symptoms, such as depression, dysarthria, and cognitive impairment.

## 6. Conclusions

In this article, we utilized gait-related features and focused on the analysis and assessment of the severity of Parkinson’s disease, via the deployment of feature selection techniques, statistical analysis and machine learning algorithms. To achieve this objective, a computational pipeline for the study of gait, based on information received from pressure sensors incorporated into a pair of insoles, has been devised, leading to an experimental protocol for the evaluation of PD motor symptoms of the lower extremities. The proposed protocol is based on results from the literature concerning the most often used tests in the field, as well as the description of pertinent items from MDS-UPDRS-Part III: Motor Examination. Both ON and OFF states have been investigated. Using MDS-UPDRS-Part III: Motor Examination, neurologists specializing in movement disorders conducted a clinical evaluation. Recordings have been obtained from PD patients, elderly and adult. Gait characteristics were extracted from the raw pressure sensor data, and a set of classification algorithms were compared for the ability to distinguish between the participant groups, the medication state of the PD patients, as well as the severity of the disease based on the MDS-UPDRS Part III total score. The models demonstrated potential in classifying PD and non-PD patients up to a rate of 88% while encouraging results have been obtained for estimation of the severity levels of the disease by achieving an up to 81% correct prediction rate during mTUG test. Our findings suggest that gait analysis can reveal valuable information for the monitoring and management of PD. It also highlights the experimental protocol’s potential for future investigations by possibly delving into different phenotypes of Parkinson’s disease, thus leading to a more precise and comprehensive analysis of phenotypic abnormalities. Further experimentation on male/female volunteers of different ages, clinical trials, as well as implementation of more advanced deep learning are considered a necessity for further and future evaluation of the proposed approach.

## Figures and Tables

**Table 1:** Details of publicly available datasets that include sensor-based gait data and focus on PD patients.

Details	Gait in Parkinson’s Disease Dataset	Daphnet Freezing of Gait Data Set	Smart-Insole Dataset
No. and Groups of participants	93 PD patients/ 73 Healthy controls	10 PD patients	8 PD patients 9 Elderly 13 Adults
Types of tests	Walking for 2 min/ Dual tasking: a subset of participants	Walking straight with 180° turn/Random walking with stops and 360° turns/Simulating ADLS	Walking straight with 180° turn/Modified Timed Up and Go Test
Test for FoG	No	Yes	No
Walking pace	Normal-self-selected	Normal-self-selected	Slow, Normal, High—self-selected
Assessment with PD scales	H and Y staging and/or UPDRS	H and Y	4 items of the MDS-UPDRS
ON and OFF medication states	Not addressed	Not addressed	Not addressed

**Table 2:** Participants details.

Group	No. of Participants	Average Age [Years]	Age Span [Years]	Height [cm]	Weight [Kg]	Gender
Adults (S)	18	50	34–59	171	76	8 Females, 10 Males
Elderly (EL)	7	70	65–78	172	80	2 Females, 5 Males
PD patients (PD)	19	63	29–74	171	78	5 Females, 14 Males

**Table 3:** The participants details and their scores on different subsets. Values are presented as Means ± Standard Deviation. Participants with dopamine continuous infusion pumps (DCIP) are reported separately.

	PD OFF State	PD ON State	PD DCIP	EL	S
No. of Participants	17	17	2	7	18
Age [years]	62 ± 11	62 ± 11	68 ± 8	70 ± 5	50 ± 6
Disease Duration [years]	10 ± 11	10 ± 11	17 ± 6	N/A	N/A
LED * [mg]	N/A	578 ± 174	1147 ± 671	N/A	N/A
Total Score MDS-UPDRS-Part III	42 ± 21	30 ± 20	33 ± 28	N/A	N/A
Total Score Control Subset **	8 ± 7	5 ± 6	9 ± 6	2 ± 2	0 ± 1

* LED stands for Levodopa Equivalent Dose, ** The Subset-Control refers to 3.9, 3.10, 3.11, 3.12, 3.13, 3.14 items of MDS-UPDRS-Part III.

**Table 4:** Results, for each different group of participants, of the estimated temporal and spatial characteristics of straight-line walking, of mTUG and WST- Slow, Normal and High tests. Values are presented as Means ± Standard Deviation.

Type of Test	WST Slow			WST Normal			WST High			mTUG		
Group of Participants	S	EL	PD	S	EL	PD	S	EL	PD	S	EL	PD
Number of Recordings *	68	28	124	68	28	128	68	28	128	68	28	124
Left Step Duration (s)	0.66 ± 0.09	0.69 ± 0.12	0.68 ± 0.13	0.60 ± 0.08	0.58 ± 0.07	0.60 ± 0.08	0.51 ± 0.07	0.49 ± 0.05	0.54 ± 0.09	0.56 ± 0.06	0.53 ± 0.07	0.57 ± 0.09
Right Step Duration (s)	0.66 ± 0.11	0.71 ± 0.12	0.69 ± 0.12	0.56 ± 0.06	0.57 ± 0.04	0.60 ± 0.08	0.52 ± 0.08	0.49 ± 0.04	0.55 ± 0.07	0.52 ± 0.06	0.54 ± 0.05	0.56 ± 0.07
Step Duration (s)	0.66 ± 0.09	0.70 ± 0.11	0.68 ± 0.12	0.58 ± 0.06	0.58 ± 0.05	0.60 ± 0.07	0.51 ± 0.08	0.49 ± 0.04	0.55 ± 0.06	0.54 ± 0.05	0.54 ± 0.05	0.57 ± 0.06
Stride Duration (s)	1.32 ± 0.18	1.40 ± 0.23	1.37 ± 0.24	1.16 ± 0.11	1.15 ± 0.09	1.20 ± 0.14	1.05 ± 0.14	0.97 ± 0.07	1.09 ± 0.12	1.08 ± 0.09	1.07 ± 0.10	1.14 ± 0.12
Steps Number	18.65 ± 1.40	18.64 ± 2.00	22.48 ± 6.52	16.00 ± 1.74	16.25 ± 1.46	19.52 ± 5.09	14.35 ± 1.52	14.39 ± 1.64	17.59 ± 5.13	15.91 ± 1.58	15.93 ± 1.76	19.54 ± 5.64
Single Support Time (s)	1.03 ± 0.24	1.21 ± 0.34	1.07 ± 0.31	0.87 ± 0.12	0.86 ± 0.08	0.90 ± 0.16	0.83 ± 0.19	0.78 ± 0.31	0.82 ± 0.10	1.14 ± 2.38	0.81 ± 0.10	0.90 ± 0.17
Double Support Time (s)	0.36 ± 0.15	0.39 ± 0.17	0.40 ± 0.16	0.35 ± 0.31	0.28 ± 0.08	0.32 ± 0.10	0.28 ± 0.10	0.23 ± 0.14	0.27 ± 0.08	0.27 ± 0.05	0.26 ± 0.06	0.28 ± 0.09
Stance Time (s)	0.84 ± 0.14	0.89 ± 0.17	0.87 ± 0.19	0.73 ± 0.10	0.71 ± 0.07	0.75 ± 0.11	0.64 ± 0.12	0.59 ± 0.06	0.66 ± 0.10	0.67 ± 0.07	0.66 ± 0.10	0.71 ± 0.10
Swing Time (s)	0.49 ± 0.06	0.52 ± 0.07	0.50 ± 0.08	0.45 ± 0.06	0.46 ± 0.05	0.47 ± 0.06	0.41 ± 0.06	0.39 ± 0.06	0.44 ± 0.04	0.43 ± 0.04	0.44 ± 0.05	0.46 ± 0.05
Single Support (%)	76.26 ± 11.64	84.63 ± 13.40	76.90 ± 12.04	72.49 ± 6.33	73.59 ± 7.26	73.80 ± 7.82	72.83 ± 6.23	72.80 ± 9.65	74.70 ± 7.12	74.96 ± 4.07	74.21 ± 5.57	73.45 ± 5.92
Double Support (%)	26.85 ± 8.65	27.37 ± 9.50	28.30 ± 7.35	26.44 ± 5.94	23.77 ± 5.70	25.85 ± 5.79	25.80 ± 6.63	23.76 ± 9.45	24.26 ± 5.20	24.15 ± 4.14	23.56 ± 4.13	23.96 ± 6.11
Stance Phase (%)	62.74 ± 3.08	62.74 ± 3.03	63.13 ± 3.53	61.84 ± 3.80	60.52 ± 3.14	61.06 ± 2.69	60.87 ± 4.01	60.17 ± 5.80	59.86 ± 3.15	60.65 ± 2.31	60.25 ± 3.28	60.39 ± 3.47
Swing Phase (%)	37.26 ± 3.08	37.26 ± 3.03	36.87 ± 3.53	38.16 ± 3.80	39.48 ± 3.14	38.94 ± 2.69	39.13 ± 4.01	39.72 ± 5.96	40.14 ± 3.15	39.35 ± 2.31	39.75 ± 3.28	39.61 ± 3.47
Gait Velocity (m/s)	0.91 ± 0.15	0.86 ± 0.19	0.78 ± 0.26	1.23 ± 0.21	1.22 ± 0.16	0.99 ± 0.25	1.52 ± 0.21	1.64 ± 0.25	1.24 ± 0.29	1.30 ± 0.18	1.34 ± 0.23	1.06 ± 0.28
Step Length (m)	0.57 ± 0.06	0.57 ± 0.06	0.50 ± 0.13	0.69 ± 0.16	0.66 ± 0.07	0.58 ± 0.14	0.75 ± 0.09	0.76 ± 0.09	0.65 ± 0.17	0.67 ± 0.07	0.67 ± 0.07	0.58 ± 0.13
Stride Length (m)	1.18 ± 0.13	1.17 ± 0.14	1.03 ± 0.29	1.39 ± 0.19	1.39 ± 0.15	1.18 ± 0.30	1.55 ± 0.18	1.58 ± 0.21	1.34 ± 0.35	1.39 ± 0.15	1.42 ± 0.18	1.19 ± 0.27
Step Frequency (steps/min)	88.67 ± 12.31	83.17 ± 12.99	85.84 ± 12.31	97.52 ± 12.26	98.75 ± 7.78	96.74 ± 10.68	110.95 ± 13.40	115.51 ± 8.26	105.30 ± 10.41	106.22 ± 10.02	105.73 ± 10.70	101.67 ± 9.56
Walk Ratio (mm/step/min)	6.62 ± 1.30	6.98 ± 1.05	5.89 ± 1.62	6.94 ± 1.32	6.74 ± 0.89	6.05 ± 1.78	6.93 ± 1.38	6.60 ± 1.08	6.14 ± 1.57	6.40 ± 0.99	6.41 ± 0.95	5.70 ± 1.31

* Number of recordings varies, since some have been excluded due technical reasons (i.e., lack of video synchronization). For PD participants ON and OFF states have been handled as separate recordings. One PD patient and one Adult (S group) were completely excluded, while some single recordings of other participants were also excluded.

**Table 5:** Results, for PD group of participants relative to ON and OFF medication state, of the estimated temporal and spatial characteristics of straight-line walking of mTUG and WST- Slow, Normal and High tests. Values are presented as Means ± Standard Deviation.

Type of Test	WST Slow		WST Normal		WST High		mTUG	
PD Medication State	OFF	ON	OFF	ON	OFF	ON	OFF	ON
Number of Recordings *	60	64	60	68	60	68	56	68
Left Step Duration (s)	0.70 ± 0.14	0.66 ± 0.11	0.61 ± 0.08	0.59 ± 0.08	0.55 ± 0.09	0.53 ± 0.08	0.58 ± 0.08	0.57 ± 0.10
Right Step Duration (s)	0.71 ± 0.13	0.66 ± 0.10	0.61 ± 0.09	0.59 ± 0.07	0.55 ± 0.07	0.55 ± 0.07	0.56 ± 0.06	0.56 ± 0.08
Step Duration (s)	0.71 ± 0.13	0.66 ± 0.10	0.61 ± 0.07	0.59 ± 0.06	0.55 ± 0.06	0.54 ± 0.06	0.57 ± 0.05	0.57 ± 0.06
Stride Duration (s)	1.41 ± 0.26	1.33 ± 0.20	1.22 ± 0.15	1.18 ± 0.12	1.10 ± 0.13	1.08 ± 0.11	1.14 ± 0.10	1.13 ± 0.13
Steps Number	22.38 ± 5.27	22.69 ± 7.40	19.10 ± 4.47	19.88 ± 5.58	16.83 ± 3.77	18.26 ± 6.04	19.14 ± 4.22	19.87 ± 6.60
Single Support Time (s)	1.10 ± 0.32	1.03 ± 0.30	0.91 ± 0.17	0.88 ± 0.15	0.84 ± 0.12	0.80 ± 0.08	0.92 ± 0.17	0.87 ± 0.16
Double Support Time (s)	0.41 ± 0.19	0.39 ± 0.12	0.32 ± 0.11	0.31 ± 0.09	0.26 ± 0.09	0.28 ± 0.07	0.28 ± 0.07	0.29 ± 0.10
Stance Time (s)	0.91 ± 0.22	0.84 ± 0.15	0.76 ± 0.12	0.73 ± 0.09	0.66 ± 0.12	0.66 ± 0.08	0.71 ± 0.09	0.71 ± 0.11
Swing Time (s)	0.52 ± 0.08	0.49 ± 0.08	0.48 ± 0.05	0.47 ± 0.07	0.45 ± 0.05	0.43 ± 0.04	0.48 ± 0.05	0.45 ± 0.05
Single Support (%)	77.55 ± 12.52	76.29 ± 11.64	73.78 ± 8.04	73.81 ± 7.68	75.70 ± 7.97	73.82 ± 6.20	73.49 ± 4.01	73.42 ± 7.16
Double Support (%)	27.69 ± 8.70	28.88 ± 5.83	25.59 ± 6.11	26.07 ± 5.54	23.34 ± 5.26	25.08 ± 5.05	23.30 ± 4.80	24.50 ± 7.00
Stance Phase (%)	63.43 ± 4.05	62.85 ± 2.98	61.03 ± 2.66	61.09 ± 2.73	59.42 ± 3.57	60.24 ± 2.69	59.76 ± 3.17	60.90 ± 3.63
Swing Phase (%)	36.57 ± 4.05	37.15 ± 2.98	38.97 ± 2.66	38.91 ± 2.73	40.58 ± 3.57	39.76 ± 2.69	40.24 ± 3.17	39.10 ± 3.63
Gait Velocity (m/s)	0.76 ± 0.27	0.80 ± 0.25	1.00 ± 0.26	0.99 ± 0.24	1.27 ± 0.30	1.21 ± 0.28	1.06 ± 0.26	1.07 ± 0.30
Step Length (m)	0.50 ± 0.14	0.50 ± 0.13	0.59 ± 0.16	0.56 ± 0.12	0.67 ± 0.19	0.63 ± 0.15	0.58 ± 0.11	0.58 ± 0.14
Stride Length (m)	1.03 ± 0.32	1.03 ± 0.27	1.21 ± 0.34	1.16 ± 0.26	1.38 ± 0.39	1.31 ± 0.32	1.19 ± 0.24	1.19 ± 0.30
Step Frequency (steps/min)	83.62 ± 12.91	87.92 ± 11.43	95.25 ± 10.82	98.05 ± 10.46	104.61 ± 10.76	105.91 ± 10.14	100.81 ± 7.84	102.38 ± 10.78
Walk Ratio (mm/step/min)	6.04 ± 1.80	5.74 ± 1.41	6.33 ± 2.14	5.81 ± 1.35	6.26 ± 1.38	6.02 ± 1.72	5.73 ± 1.15	5.68 ± 1.44

* Number of recordings varies, since some have been excluded due technical reasons (i.e., lack of video synchronization). One PD patient was excluded, while some single recordings of other participants were also excluded.

**Table 6:** Results of the estimated temporal and spatial characteristics, for PD group of participants relative to the Severity Levels and MDS-UPDRS- Part III, of straight-line walking of WST-Slow-Normal-High and mTUG tests. Values are presented as Means ± Standard Deviation.

Type of Test	WST-Slow		WST-Normal		WST-High		mTUG	
Severity Level *	Mild	Moderate	Mild	Moderate	Mild	Moderate	Mild	Moderate
Number of Recordings **	64	60	68	60	68	60	64	60
Left Step Duration (s)	0.67 ± 0.1	0.69 ± 0.15	0.59 ± 0.07	0.61 ± 0.09	0.53 ± 0.1	0.55 ± 0.06	0.56 ± 0.09	0.59 ± 0.09
Right Step Duration (s)	0.67 ± 0.11	0.70 ± 0.14	0.59 ± 0.07	0.61 ± 0.09	0.56 ± 0.08	0.54 ± 0.06	0.56 ± 0.06	0.57 ± 0.08
Step Duration (s)	0.67 ± 0.09	0.70 ± 0.14	0.59 ± 0.05	0.61 ± 0.08	0.55 ± 0.06	0.55 ± 0.05	0.56 ± 0.05	0.58 ± 0.06
Stride Duration (s)	1.34 ± 0.19	1.40 ± 0.28	1.18 ± 0.11	1.22 ± 0.16	1.09 ± 0.13	1.09 ± 0.1	1.12 ± 0.1	1.15 ± 0.13
Steps Number	19.00 ± 2.95	26.20 ± 7.22	17.12 ± 2.37	22.23 ± 5.93	15.32 ± 2.11	20.17 ± 6.24	16.98 ± 2.38	22.27 ± 6.75
Single Support Time (s)	1.06 ± 0.26	1.07 ± 0.37	0.88 ± 0.12	0.92 ± 0.20	0.80 ± 0.07	0.83 ± 0.13	0.88 ± 0.13	0.91 ± 0.2
Double Support Time (s)	0.36 ± 0.09	0.44 ± 0.2	0.30 ± 0.08	0.33 ± 0.11	0.28 ± 0.09	0.26 ± 0.07	0.27 ± 0.09	0.29 ± 0.09
Stance Time (s)	0.84 ± 0.14	0.91 ± 0.22	0.73 ± 0.08	0.77 ± 0.13	0.66 ± 0.11	0.66 ± 0.08	0.70 ± 0.09	0.73 ± 0.11
Swing Time (s)	0.52 ± 0.08	0.49 ± 0.08	0.47 ± 0.05	0.48 ± 0.07	0.43 ± 0.03	0.45 ± 0.05	0.46 ± 0.05	0.47 ± 0.05
Single Support (%)	77.83 ± 11.15	75.91 ± 12.95	74.64 ± 7.49	72.85 ± 8.14	74.19 ± 6.61	75.27 ± 7.67	73.62 ± 5.58	73.28 ± 6.32
Double Support (%)	26.52 ± 4.72	30.21 ± 9.04	25.30 ± 4.71	26.47 ± 6.80	24.91 ± 4.96	23.53 ± 5.41	23.45 ± 5.72	24.49 ± 6.51
Stance Phase (%)	61.77 ± 2.64	64.58 ± 3.8	60.76 ± 2.40	61.41 ± 2.96	60.24 ± 3.05	59.42 ± 3.22	59.96 ± 3.37	60.84 ± 3.54
Swing Phase (%)	38.23 ± 2.64	35.42 ± 3.8	39.24 ± 2.40	38.59 ± 2.96	39.76 ± 3.05	40.58 ± 3.22	40.04 ± 3.37	39.16 ± 3.54
Gait Velocity (m/s)	0.90 ± 0.23	0.65 ± 0.22	1.11 ± 0.18	0.86 ± 0.24	1.38 ± 0.2	1.07 ± 0.29	1.20 ± 0.21	0.92 ± 0.28
Step Length (m)	0.57 ± 0.11	0.43 ± 0.11	0.64 ± 0.12	0.51 ± 0.13	0.72 ± 0.16	0.56 ± 0.14	0.64 ± 0.09	0.51 ± 0.13
Stride Length (m)	1.18 ± 0.27	0.87 ± 0.23	1.31 ± 0.27	1.04 ± 0.27	1.50 ± 0.33	1.16 ± 0.29	1.32 ± 0.2	1.04 ± 0.27
Step Frequency (steps/min)	87.12 ± 11.74	84.48 ± 12.84	97.70 ± 9.02	95.65 ± 12.27	105.00 ± 10.59	105.64 ± 10.29	102.52 ± 8.4	100.76 ± 10.66
Walk Ratio (mm/step/min)	6.63 ± 1.62	5.09 ± 1.17	6.63 ± 1.80	5.39 ± 1.51	6.78 ± 1.43	5.41 ± 1.39	6.27 ± 1.08	5.10 ± 1.27

* There is no participation clustered as normal level. One PD patient rated as severe level was recorded, however he was excluded from the analysis since he need support to complete all the tests. ** Number of recordings varies, since some have been excluded due technical reasons (i.e., lack of video synchronization). For PD participants ON and OFF states have been handled as separate recordings. One PD patient and one Adult (S group) were completely excluded, while some single recordings of other participants were also excluded.

**Table 7:** Features affected in a statistically significant manner (*p* < 0.05), elaborating WST Slow-Normal-High and mTUG tests, relevant to the ON/OFF medication state and MDS-UPDRS-Part III ratings in relation to the severity levels.

Statistical Significance	*p*-Values							
Related Class	Medication State ON/OFF				MDS-UPDRS-Part III/ Severity Levels			
Type of Test	WST Slow	WST Normal	WST High	mTUG	WST Slow	WST Normal	WST High	mTUG
Left Step Duration (s)	**0.010**	0.923	0.665	0.257	0.154	0.276	0.300	0.679
Right Step Duration (s)	**0.006**	0.923	0.302	0.929	**0.049**	**0.016**	0.783	0.614
Step Duration (s)	**0.003**	0.274	0.216	0.477	0.055	0.110	0.349	0.959
Stride Duration (s)	**0.004**	0.823	0.213	0.486	0.055	**0.043**	0.415	0.891
Steps Number	**0.018**	0.073	**0.000**	**0.021**	**0.018**	**0.001**	0.434	0.132
Single Support Time (s)	**0.010**	0.562	**0.000**	0.158	**0.011**	**0.004**	0.114	0.193
Double Support Time (s)	0.454	0.677	**0.023**	0.651	0.504	0.753	0.124	0.915
Stance Time (s)	**0.007**	0.829	0.894	0.943	**0.048**	0.149	0.711	0.660
Swing Time (s)	**0.000**	**0.003**	**0.001**	**0.040**	**0.005**	**0.000**	**0.003**	0.217
Single Support (%)	0.074	0.757	**0.003**	0.328	**0.061**	0.447	0.593	0.254
Double Support (%)	0.139	0.247	**0.000**	0.809	0.693	0.169	**0.005**	0.871
Stance Phase (%)	0.458	0.289	**0.013**	0.290	0.611	**0.006**	**0.041**	0.811
Swing Phase (%)	0.683	0.222	**0.010**	0.345	0.611	**0.006**	**0.041**	0.811
Gait Velocity (m/s)	0.792	**0.026**	0.152	**0.003**	**0.010**	**0.004**	0.334	**0.036**
Step Length (m)	0.136	**0.043**	0.056	**0.009**	**0.031**	**0.026**	0.679	**0.082**
Stride Length (m)	0.179	**0.037**	0.091	**0.012**	**0.055**	**0.042**	0.630	**0.060**
Step Frequency (steps/min)	**0.020**	**0.000**	0.053	0.926	**0.020**	0.543	**0.068**	0.906
Walk Ratio (mm/step/min)	**0.008**	**0.042**	0.150	**0.032**	0.337	0.382	0.846	0.262

**Table 8:** Classification results, reported as accuracy, elaborating the WST Slow-Normal-High and mTUG tests. The best estimator for each classification approach is highlighted.

Classification	PD-nonPD				PD-EL-S				Medication State ON/OFF				MDS-UPDRS: Part III Severity Levels			
Type of Test	WST Slow	WST Normal	WST High	mTUG	WST Slow	WST Normal	WST High	mTUG	WST Slow	WST Normal	WST High	mTUG	WST Slow	WST Normal	WST High	mTUG
AdaBoost	0.82	**0.73**	**0.83**	**0.85**	0.70	0.62	0.50	**0.66**	0.58	0.54	**0.69**	0.65	**0.77**	0.68	0.58	**0.81**
Extra Trees	0.85	**0.73**	0.76	0.80	0.74	0.64	0.64	0.57	**0.62**	**0.64**	0.58	**0.73**	0.65	0.68	0.62	0.69
Random Forest	**0.88**	**0.73**	0.71	0.75	**0.77**	0.60	0.70	0.57	**0.62**	0.61	0.62	0.62	0.73	0.57	0.62	0.73

## References

[1] Dorsey E.R., Sherer T., Okun M.S., Bloemd B.R. (2018). The emerging evidence of the parkinson pandemic. J. Park. Dis..

[2] Kouli A., Torsney K.M., Kuan W.-L., Stoker T.B., Greenland J.C. (2018). Parkinson’s Disease: Pathogenesis and Clinical Aspects.

[3] Copas A.N.M., McComish S.F., Fletcher J.M., Caldwell M.A. (2021). The pathogenesis of parkinson’s disease: A complex interplay between astrocytes, microglia, and T lymphocytes?. Front. Neurol..

[4] Emamzadeh F.N., Surguchov A. (2018). Parkinson’s disease: Biomarkers, treatment, and risk factors. Front. Neurosci..

[5] Tolosa E., Garrido A., Scholz S.W., Poewe W. (2021). Challenges in the diagnosis of Parkinson’s disease. Lancet Neurol..

[6] Aarsland D., Batzu L., Halliday G.M., Geurtsen G.J., Ballard C., Chaudhuri K.R., Weintraub D. (2021). Parkinson disease-associated cognitive impairment. Nat. Rev. Dis. Prim..

[7] Moya-Galé G., Levy E.S. (2019). Parkinson’s disease-associated dysarthria: Prevalence, impact and management strategies. Res. Rev. Park..

[8] Hallett M. (2012). Parkinson’s disease tremor: Pathophysiology. Park. Relat. Disord..

[9] Gandhi K.R., Saadabadi A. Levodopa (L-Dopa). https://www.ncbi.nlm.nih.gov/books/NBK482140/.

[10] Sharma V.D., Patel M., Miocinovic S. (2020). Surgical treatment of parkinson’s disease: Devices and lesion approaches. Neurotherapeutics.

[11] Marsili L., Rizzo G., Colosimo C. (2018). Diagnostic criteria for Parkinson’s disease: From James Parkinson to the concept of prodromal disease. Front. Neurol..

[12] Goetz C.G., Fahn S., Martinez-Martin P. (2008). The MDS-sponsored Revision of the Unified Parkinson’s Disease Rating Scale. J. Mov. Disord..

[13] Hoehn M.M., Yahr M.D. (2001). Parkinsonism: Onset, progression, and mortality 1967. Neurology.

[14] Schlachetzki J.C.M., Barth J., Marxreiter F., Gossler J., Kohl Z., Reinfelder S., Gassner H., Aminian K., Eskofier B.M., Winkler J. (2017). Wearable sensors objectively measure gait parameters in Parkinson’s disease. PLoS ONE.

[15] Subramaniam S., Majumder S., Faisal A.I., Deen M.J. (2022). Insole-based systems for health monitoring: Current solutions. Sensors.

[16] Chatzaki C., Skaramagkas V., Tachos N., Christodoulakis G., Maniadi E., Kefalopoulou Z., Fotiadis D., Tsiknakis M. (2021). The smart-insole dataset: Gait analysis using wearable sensors with a focus on elderly and Parkinson’s patients. Sensors.

[17] Lu M., Zhao Q., Poston K.L., Sullivan E.V., Pfefferbaum A., Shahid M., Katz M., Kouhsari L.M., Schulman K., Milstein A. (2021). Quantifying Parkinson’s disease motor severity under uncertainty using MDS-UPDRS videos. Med. Image Anal..

[18] Mandal I., Sairam N., Mandal I., Sairam N. (2014). New machine-learning algorithms for prediction of Parkinson’s disease. Int. J. Syst. Sci..

[19] Ahlrichs C., Lawo M. (2013). Parkinson’s disease motor symptoms in machine learning: A review. Health Inform. Int. J..

[20] Skaramagkas V., Andrikopoulos G., Kefalopoulou Z., Polychronopoulos P. (2021). A study on the essential and parkinson’s arm tremor classification. Signals.

[21] Skaramagkas V., Andrikopoulos G., Kefalopoulou Z., Polychronopoulos P. (2020). Towards differential diagnosis of essential and parkinson’s tremor via machine learning. Proceedings of the 2020 28th Mediterranean Conference on Control and Automation (MED), Saint-Raphaël.

[22] Papadopoulos A., Kyritsis K., Klingelhoefer L., Bostanjopoulou S., Chaudhuri K.R., Delopoulos A. Detecting parkinsonian tremor from IMU data collected in-the-wild using deep multiple-instance learning. https://zenodo.org/record/3519213.

[23] Goschenhofer J., Pfister F.M.J., Yuksel K.A., Bischl B., Fietzek U., Thomas J. (2019). Wearable-based parkinson’s disease severity monitoring using deep learning. Lect. Notes Comput. Sci..

[24] Ibrahim A., Zhou Y., Jenkins M.E., Trejos A.L., Naish M.D. (2020). The design of a parkinson’s tremor predictor and estimator using a hybrid convolutional-multilayer perceptron neural network. Proceedings of the 2020 42nd Annual International Conference of the IEEE Engineering in Medicine & Biology Society (EMBC).

[25] Hobert M.A., Maetzler W., Aminian K., Chiari L. (2014). Technical and clinical view on ambulatory assessment in Parkinson’s disease. Acta Neurol. Scand..

[26] Dewey D.C., Miocinovic S., Bernstein I., Khemani P., Dewey R.B., Querry R., Chitnis S., Dewey R.B. (2014). Automated gait and balance parameters diagnose and correlate with severity in Parkinson disease. J. Neurol. Sci..

[27] Kyrarini M., Wang X., Graser A. (2015). Comparison of vision-based and sensor-based systems for joint angle gait analysis. Proceedings of the 2015 IEEE International Symposium on Medical Measurements and Applications (MeMeA) Proceedings.

[28] Moro M., Marchesi G., Hesse F., Odone F., Casadio M. (2022). Markerless vs. marker-based gait analysis: A proof of concept study. Sensors.

[29] Goldberger A.L., Amaral L.A., Glass L., Hausdorff J.M., Ivanov P.C., Mark R.G., Mietus J.E., Moody G.B., Peng C.K., Stanley H.E. (2000). PhysioBank, PhysioToolkit, and PhysioNet: Components of a new research resource for complex physiologic signals. circulation.

[30] Bachlin M., Plotnik M., Roggen D., Maidan I., Hausdorff J.M., Giladi N., Troster G. (2010). Wearable assistant for Parkinsons disease patients with the freezing of gait symptom. IEEE Trans. Inf. Technol. Biomed..

[31] Zanardi A.P.J., da Silva E.S., Costa R.R., Passos-Monteiro E., dos Santos I.O., Kruel L.F.M., Peyré-Tartaruga L.A. (2021). Gait parameters of Parkinson’s disease compared with healthy controls: A systematic review and meta-analysis. Sci. Rep..

[32] Braun B., Veith N.T., Hell R., Döbele S., Roland M., Rollmann M., Holstein J.H., Pohlemann T. (2015). Validation and reliability testing of a new, fully integrated gait analysis insole. J. Foot Ankle Res..

[33] Stöggl T., Martiner A. (2017). Validation of Moticon’s OpenGo sensor insoles during gait, jumps, balance and cross-country skiing specific imitation movements. J. Sports Sci..

[34] Kakarla T.P., Varma K.A., Preejith S.P., Joseph J., Sivaprakasam M. (2019). Accuracy Enhancement of Total Force by Capacitive Insoles. Proceedings of the 2019 IEEE International Symposium on Medical Measurements and Applications (MeMeA).

[35] Moticon-SCIENCE. https://www.moticon.de/.

[36] Bloem B.R., Hausdorff J.M., Visser J.E., Giladi N. (2004). Falls and freezing of Gait in Parkinson’s disease: A review of two interconnected, episodic phenomena. Mov. Disord..

[37] Brognara L., Palumbo P., Grimm B., Palmerini L. (2019). Assessing gait in Parkinson’s disease using wearable motion sensors: A systematic review. Diseases.

[38] Podsiadlo D., Richardson S. (1991). The timed ‘Up & Go’: A test of basic functional mobility for frail elderly persons. J. Am. Geriatr. Soc..

[39] Herman T., Giladi N., Hausdorff J.M. (2011). Properties of the ‘Timed Up and Go’ test: More than meets the eye. Gerontology.

[40] McGrath D., Greene B.R., Doheny E.P., McKeown D.J., de Vito G., Caulfield B. (2011). Reliability of quantitative TUG measures of mobility for use in falls risk assessment. Proceedings of the Annual International Conference of the IEEE Engineering in Medicine and Biology Society.

[41] Mariani B., Jiménez M.C., Vingerhoets F.J.G., Aminian K. (2013). On-shoe wearable sensors for gait and turning assessment of patients with Parkinson’s disease. IEEE Trans. Biomed. Eng..

[42] Snijders A.H., Weerdesteyn V., Hagen Y.J., Duysens J., Giladi N., Bloem B.R. (2010). Obstacle avoidance to elicit freezing of gait during treadmill walking. Mov. Disord..

[43] Jacobs J.V., Horak F.B., Tran V.K., Nutt J.G. (2006). Multiple balance tests improve the assessment of postural stability in subjects with Parkinson’s disease. J. Neurol. Neurosurg. Psychiatry.

[44] Brauer S.G., Woollacott M.H., Lamont R., Clewett S., O’Sullivan J., Silburn P., Mellick G.D., Morris M.E. (2011). Single and dual task gait training in people with Parkinson’s Disease: A protocol for a randomised controlled trial. BMC Neurol..

[45] Ziegler K., Schroeteler F., Ceballos-Baumann A.O., Fietzek U.M. (2010). A new rating instrument to assess festination and freezing gait in Parkinsonian patients. Mov. Disord..

[46] Kluge F., Gaßner H., Hannink J., Pasluosta C., Klucken J., Eskofier B.M. (2017). Towards mobile gait analysis: Concurrent validity and test-retest reliability of an inertial measurement system for the assessment of spatio-temporal gait parameters. Sensors.

[47] Combs S.A., Diehl M.D., Filip J., Long E. (2014). Short-distance walking speed tests in people with Parkinson disease: Reliability, responsiveness, and validity. Gait Posture.

[48] Martínez-Martín P., Rodriguez-Blazquez C., Alvarez M., Arakaki T., Arillo V.C., Chaná P., Fernández W., Garretto N., Castrillo J.C.M., Rodríguez-Violante M. (2015). Parkinson’s disease severity levels and MDS-Unified Parkinson’s Disease Rating Scale. Park. Relat. Disord..

[49] Kefalopoulou Z., Chatzaki V., Skaramagkas C., Chroni E., Tachos N., Fotiadis D.I., Tsiknakis M. (2022). Pressure Sensor Insole Gait Assessment for Parkinson’s Disease Patients: A Pilot Study [Abstract]. Movement Disorder 2022 International Congress.

[50] Kasović M., Štefan L., Štefan A. (2021). Normative data for gait speed and height norm speed in ≥ 60-year-old men and women. Clin. Interv. Aging.

[51] Peel N.M., Kuys S.S., Klein K. (2013). Gait speed as a measure in geriatric assessment in clinical settings: A systematic review. J. Gerontol. Ser. A Biol. Sci. Med. Sci..

[52] Paker N., Bugdayci D., Goksenoglu G., Demircioğlu D.T., Kesiktas N., Ince N. (2015). Gait speed and related factors in parkinson’s disease. J. Phys. Ther. Sci..

[53] Rota V., Perucca L., Simone A., Tesio L. (2011). Walk ratio (step length/cadence) as a summary index of neuromotor control of gait: Application to multiple sclerosis. Int. J. Rehabil. Res..

[54] Wu A.R., Simpson C.S., van Asseldonk E.H.F., van der Kooij H., Ijspeert A.J. (2019). Mechanics of very slow human walking. Sci. Rep..

[55] Murakami R., Otaka Y. (2017). Estimated lower speed boundary at which the walk ratio constancy is broken in healthy adults. J. Phys. Ther. Sci..

[56] Vila M.H., Pérez R., Mollinedo I., Cancela J.M. (2021). Analysis of gait for disease stage in patients with parkinson’s disease. Int. J. Environ. Res. Public Health.

[57] Kwon K.Y., Lee H.M., Kang S.H., Pyo S.J., Kim H.J., Koh S.B. (2017). Recuperation of slow walking in de novo Parkinson’s disease is more closely associated with increased cadence, rather than with expanded stride length. Gait Posture.

[58] Agrawal P., Abutarboush H.F., Ganesh T., Mohamed A.W. (2021). Metaheuristic algorithms on feature selection: A survey of one decade of research (2009–2019). IEEE Access.

[59] Rácz A., Bajusz D., Héberger K. (2019). Multi-level comparison of machine learning classifiers and their performance metrics. Molecules.

[60] Gholamy A., Kreinovich V., Kosheleva O. (2018). Why 70/30 or 80/20 Relation between Training and Testing Sets: A Pedagogical Explanation. Departmental Technical Reports (CS). Feburary. https://scholarworks.utep.edu/cs_techrep/1209/.

[61] Kuhn M., Johnson K. (2013). Over-fitting and model tuning. Applied Predictive Modeling.

[62] Curtze C., Nutt J.G., Carlson-Kuhta P., Mancini M., Horak F.B. (2015). Levodopa is a double-edged sword for balance and gait in people with parkinson’s disease. Mov. Disord..

[63] Cabeleira M.E.P., Pagnussat A.S., do Pinho A.S., Asquidamini A.C.D., Freire A.B., Pereira B.T., de Mello Rieder C.R., Schifino G.P., Fornari L.H.T., Junior N.D.S. (2019). Impairments in gait kinematics and postural control may not correlate with dopamine transporter depletion in individuals with mild to moderate Parkinson’s disease. Eur. J. Neurosci..

[64] Oliveira J.A., Bazán P.R., Oliveira C.E.N., Treza R.D.C., Hondo S.M., Angeles E.L., Bernardo C., Oliveira L.D.S., Carvalho M.D.J., Lima-Pardini A.C. (2021). The effects of levodopa in the spatiotemporal gait parameters are mediated by self-selected gait speed in Parkinson’s disease. Eur. J. Neurosci..

[65] Loh H.W., Hong W., Ooi C.P., Chakraborty S., Barua P.D., Deo R.C., Soar J., Palmer E.E., Acharya U.R. (2021). Application of Deep Learning Models for Automated Identification of Parkinson’s Disease: A Review (2011–2021). Sensors.

[66] Vasquez-Correa J.C., Arias-Vergara T., Orozco-Arroyave J.R., Eskofier B.M., Klucken J., Noth E. (2019). Multimodal Assessment of Parkinson’s Disease: A Deep Learning Approach. IEEE J. Biomed. Health Inform..

